# 204. Clinical and Epidemiologic Review of Capnocytophaga Infections Identified at a Public Health Reference Laboratory – California, 2005 – 2021

**DOI:** 10.1093/ofid/ofad500.277

**Published:** 2023-11-27

**Authors:** Emily Kelly, Rebecca Campagna, Duc Vugia, Curtis L Fritz

**Affiliations:** University of California, San Francisco, San Francisco, California; Infectious Diseases Branch, Division of Communicable Disease Control, Center for Infectious Diseases, California Department of Public Health, Sacramento, California; California Department of Public Health, Richmond, CA; Infectious Diseases Branch, Division of Communicable Disease Control, Center for Infectious Diseases, California Department of Public Health, Sacramento, California

## Abstract

**Background:**

*Capnocytophaga* spp. are bacteria commonly found in the mouths of dogs and cats, which can infect people through bites or other contact with saliva. This study describes the epidemiology and clinical features of patients from whom *Capnocytophaga* spp. were isolated from clinical specimens at the California State Microbial Diseases Laboratory (MDL) over a 17-year period.

**Methods:**

All MDL reports of *Capnocytophaga* spp. isolated from human patients between 2005 and 2021, including specimen submission forms and accompanying medical records, were reviewed. Data from these records were used to identify patients in datasets of statewide hospital patient discharge (PD) and emergency department (ED) admissions when possible. Clinical and other information in the PD and ED databases were used to confirm and supplement information from laboratory records.

**Results:**

*Capnocytophaga* spp. were isolated in 48 specimens from 47 patients. The median age of patients was 68 years (range 12 to 88 years), and 57% were male. *C. canimorsus* represented 83% of the *Capnocytophaga* spp. identified. *Capnocytophaga* spp. were most frequently isolated from blood (88%), followed by cerebrospinal fluid (4%) and synovial fluid (4%). The most common case presentation was sepsis (45%), followed by skin and soft tissue infection (13%). Records for 45% of patients indicated at least one comorbidity associated with immunosuppression, including current malignancy (19%), diabetes mellitus (17%), alcohol use disorder (6%), and history of splenectomy (6%). Six records (13%) reported an animal bite (five dog bites, one cat bite).

**Conclusion:**

This study documented *Capnocytophaga* spp. infection causing rare, severe disease in older and immunocompromised persons, often without report of an animal bite. This vulnerable group should be made aware of the risk of *Capnocytophaga* spp. infection from contact with cats and dogs and counseled to seek prompt medical care for any signs of infection, even if no known bite occurred.

**Disclosures:**

**All Authors**: No reported disclosures

